# Insect ectoparasites from wild passerine birds in the Azores Islands

**DOI:** 10.1051/parasite/2020063

**Published:** 2020-11-20

**Authors:** Lucie Oslejskova, Sarka Kounkova, Daniel R. Gustafsson, Roberto Resendes, Pedro Rodrigues, Ivan Literak, Oldrich Sychra

**Affiliations:** 1 Department of Biology and Wildlife Diseases, Faculty of Veterinary Hygiene and Ecology, University of Veterinary and Pharmaceutical Sciences Brno Palackeho tr. 1946/1 61242 Brno Czech Republic; 2 Guangdong Key Laboratory of Animal Conservation and Resources, Guangdong Public Laboratory of Wild Animal Conservation and Utilization, Guangdong Institute of Zoology 105 Xingang West Road Haizhu District Guangzhou 510260 China; 3 CIBIO, Centro de Investigação em Biodiversidade e Recursos Genéticos, InBIO Laboratório Associado, Polo dos Açores, Universidade dos Açores 9501-801 Ponta Delgada Portugal; 4 Instituto de Patologia Animal, Facultad de Ciencias Veterinarias, Universidad Austral de Chile Isla Teja 5090000 Valdivia Chile

**Keywords:** Chewing lice, Phthiraptera, flea, Siphonaptera, louse-fly, Hippoboscidae, Passeriformes, phoresy

## Abstract

A total of 266 wild passerine birds (Passeriformes) representing eight species and nine subspecies from three islands of the Archipelago of the Azores were examined for ectoparasites. Two species of louse-flies *Ornithomya avicularia* and *Ornithoica turdi* (Diptera: Hippoboscidae), three species of fleas *Ceratophyllus gallinae*, *Ceratophyllus* sp. and *Dasypsyllus gallinulae* (Siphonaptera: Ceratophyllidae), and 11 species of chewing lice belonging to the genera *Menacanthus, Myrsidea* (Phthiraptera: Menoponidae), *Ricinus* (Phthiraptera: Ricinidae), *Brueelia*, *Guimaraesiella*, *Philopterus*, *Sturnidoecus* and *Turdinirmus* (Phthiraptera: Philopteridae) were recorded. At least one species of ectoparasite was found on 114 birds of six species. *Guimaraesiella tovornikae* and *Myrsidea sylviae* from *Sylvia atricapilla* are redescribed. Records of *Ceratophyllus* sp. and *Sturnidoecus* sp. from *Turdus merula* represent new parasite-host associations. Phoresy of *Guimaraesiella amsel* on *Ornithoica turdi* was also found. Parasitological parameters such as prevalence, intensity and abundance and geographic distribution of recorded ectoparasites are provided.

## Abstract (fr)

Un total de 266 passereaux sauvages (Passeriformes) de trois îles de l’archipel des Açores, représentant huit espèces et neuf sous-espèces, ont été examinés pour la recherche d’ectoparasites. Deux espèces de mouches-araignées, *Ornithomya avicularia* et *Ornithoica turdi* (Diptera : Hippoboscidae), trois espèces de puces, *Ceratophyllus gallinae*, *Ceratophyllus* sp. et *Dasypsyllus gallinulae* (Siphonaptera : Ceratophyllidae), et onze espèces de mallophages appartenant aux genres *Menacanthus, Myrsidea* (Phthiraptera : Menoponidae), *Ricinus* (Phthiraptera : Ricinidae), *Brueelia*, *Guimaraesiella*, *Philopterus*, *Sturnidoecus* et *Turdinirmus* (Phthiraptera : Philopteridae) sont signalées. Au moins une espèce d’ectoparasite a été trouvée sur 114 oiseaux de six espèces. *Guimaraesiella tovornikae* et *Myrsidea sylviae*, de *Sylvia atricapilla,* sont redécrits. Les signalements de *Ceratophyllus* sp. et *Sturnidoecus* sp. chez *Turdus merula* représentent de nouvelles associations parasites-hôtes. La phorésie de *Guimaraesiella amsel* sur *Ornithoica turdi* a également été trouvée. Les paramètres parasitologiques des ectoparasites signalés, tels que la prévalence, l'intensité, l'abondance et la répartition géographique, sont fournis.

## Introduction

A total of 414 species of birds have been recorded in the Azores, including endemic resident birds, introduced species and escapees from captivity, as well as migrating non-breeding species and occasional vagrants from either the European or American continents [5, 59]. Only 37 bird species and subspecies regularly breed and seven other species occasionally nest in the Azores, including 16 (36%) species and subspecies of passerine birds: 10 endemic species and subspecies: *Fringilla coelebs moreletti* Pucheran, 1859, *Motacilla cinerea patriciae* Vaurie, 1957, *Pyrrhula murina* Godman, 1866, *Regulus regulus azoricus* Seebohm, 1883, *Regulus regulus inermis* Murphy & Chapin, 1929, *Regulus regulus sanctaemariae* Vaurie, 1954, *Serinus canaria* (Linnaeus, 1758), *Sturnus vulgaris granti* Hartert, 1903, *Sylvia atricapilla gularis* Alexander, 1898 and *Turdus merula azorensis* Hartert, 1905; two native species: *Erithacus rubecula rubecula* (Linnaeus, 1758) and *Oenanthe oenanthe leucorhoa* (Gmelin, 1789); and four introduced species: *Carduelis carduelis parva* Tschusi, 1901, *Chloris chloris aurantiiventris* (Cabanis, 1851), *Estrilda astrild* (Linnaeus, 1758), and *Passer domesticus domesticus* (Linnaeus, 1758). In spite of the geographical location of the Azorean islands in the middle of the Atlantic Ocean, and of the prevailing westerly winds, none of the species that breed in the Azores has a Nearctic origin [59]. However, many Nearctic vagrants are recorded in the Archipelago each year [1].

Few bird parasites have been recorded from the Azores. Only 4 of the 15 species of fleas (Siphonaptera) recorded in the archipelago have been reported from birds and all of them only in São Miguel island: *Ceratophyllus* (*Ceratophyllus*) *gallinae gallinae* (Schrank, 1803), *Ceratophyllus* (*Ceratophyllus*) *hirundinis* (Curtis, 1826), *Ceratophyllus* (*Monopsyllus*) *sciurorum sciurorum* (Schrank, 1803), and *Dasypsyllus gallinulae gallinulae* (Dale, 1878) [8, 33]. Similarly, only two species of louse-flies (Hippoboscidae) have been recorded: *Hippobosca equina* Linnaeus, 1758 – a parasite of horses, and *Ornithomya chloropus* Bergroth, 1901 – a species associated with various species of passerine birds [37, 65, 69]. Despite the relatively high number of potential hosts, only 19 species of chewing lice (Phthiraptera) have been reported from the Azores [51]. These reports concern mainly marine birds, and only two species of chewing lice have been reported from passerine hosts in the Azores [34].

In this paper, we expand the knowledge of ectoparasites of passerine birds from the Archipelago of the Azores [34, 35, 63]. The aims of this paper are to: (1) present new data on the species distribution of insect ectoparasites found on passerine birds in the Azores; (2) include information on their parasitological parameters; and (3) redescribe *Guimaraesiella tovornikae* (Balát, 1981) and *Myrsidea sylviae* Sychra & Literak, 2008.

## Materials and methods

The Macaronesian archipelago of the Azores is situated in the Atlantic between 36°55′ and 39°43′N and 24°46′ and 31°16′W, and comprises nine islands. The total surface area of these islands is about 2300 km^2^ and the islands stretch more than 600 km from northwest to southeast. The nearest point on the mainland is Cabo da Roca in Portugal, which is 1408 km east of Santa Maria Island. In 2013, a total of 266 resident passerine birds were mist-netted on the Azores. Birds were captured at various sites on each of the three islands explored. Call playback was used to bring birds to the net. Birds were identified using a field guide [16] and ringed. Totals of 107, 84, and 75 birds from São Miguel (14–19 April 2013), Santa Maria (18–21 September 2013), and Graciosa (21–24 September 2013), respectively, were examined. Ectoparasites were collected by visual examination and using the fumigation chamber method, using chloroform as a fumigant with visual search of the head [18]. Birds were released after examination. Ectoparasites were stored in 96% ethanol. Chewing lice and fleas were subsequently slide-mounted in Canada balsam as permanent slides, following the technique in Palma [50]. Identification of the lice was based on Price [56], Gustafsson and Bush [24], Sychra and Literak [71], Rheinwald [58] and Najer et al. [46]. Identification of the louse-flies and fleas was based on Chvala [15] and Rosicky [66]. The taxonomy of birds follows Clements et al. [19].

In the following redescriptions, all the morphological descriptions and characters, as well as the terminology of chaetotaxy were taken from or follow those from Clay [17] and Sychra and Literak [71] for *Myrsidea* and Gustafsson and Bush [24] for *Guimaraesiella* and *Sturnidoecus*; all dimensions are given in millimetres; abbreviations for setae and measured features are: *ads* = *anterior dorsal seta*; *aps = accessory post-spiracular seta*; *dhs = dorsal head seta*; *ps = paratergal seta*; *psps = principal post-spiracular seta*; *pst1–pst2 = parameral setae 1–2*; *s1–s4 = aster setae length (setae are counted from the longest inner seta to the shortest outer one)*; *ss = sutural seta*; *sts = sternal seta*; *tps = tergal posterior seta*; *vms = vulval marginal seta*; *vos = vulval oblique seta*; *vss = vulval submarginal seta*; ANW = female anus width; AW = abdomen width [at level of segment IV (for *Myrsidea*) or V (for *Guimaraesiella* and *Sturnidoecus*)]; GSL = male genital sac sclerite length; GW = male genitalia width; HL = head length (at midline); HW = head width (at temples); MW = metathorax width; ParL = paramere length; POW = preocular width; PTW = pterothorax width; PW = prothorax width; TL = total length. The specimens examined are deposited at the Moravian Museum, Brno, Czech Republic (MMBC); in addition, we examined some specimens from the Slovenian Museum of Natural History, Ljubljana, Slovenia (PMSL).

Parasitological parameters were counted as in Sychra et al. [73]. We used the following categories to designate the infestation on passerine hosts: very light infestation (1–10 lice per bird); light infestation (11–20 lice); medium infestation (21–30 lice); heavy infestation (31–50 lice); very heavy infestation (51–100 lice); extremely heavy infestation (>100 lice). For statistical analyses, Fisher’s exact test (for prevalences) and bootstrap 2-sample *t*-test (for intensities and abundances) were used. Calculations were made in Quantitative Parasitology 3.0 [68].

## Results

A total of 266 passerine birds representing eight species and nine subspecies were sampled on three Azores Islands (Table 1). No insect ectoparasite was found on *Carduelis carduelis* or *Serinus canaria*.

**Table 1:** List of hosts and their insect ectoparasites. Abbreviation: Prev. = prevalence = number of birds parasitized/number of birds examined, Ny = nymphs, C = Ceratophyllidae, H = Hippoboscidae, M = Menoponidae, P = Philopteridae, R = Ricinidae; ^FM^ = gynandromorphs.

Bird species Ectoparasite family/species	São Miguel (April)				Santa Maria (September)				Graciosa (September)			
	Prev.	♂	♀	Ny	Prev.	♂	♀	Ny	Prev.	♂	♀	Ny
Family Fringillidae												
*Carduelis carduelis parva* Tschusi, 1901	0/2	–	–	–	–	–	–	–	–	–	–	–
*Fringilla coelebs moreletti* Pucheran, 1859												
C/*Dasypsyllus gallinulae* (Dale, 1878)**	3/27	0	3	–	0/14	–	–	–	2/61	1	1	–
H/*Ornithomya avicularia* (Linnaeus, 1758)**	0/27	–	–	–	0/14	–	–	–	4/61	1	5	–
H/*Ornithoica turdi* (Olivier in Latreille, 1811)**	0/27	–	–	–	0/14	–	–	–	7/61	2	6	–
M/*Menacanthus eurysternus* (Burmeister, 1838)**	1/27	0	0	8	0/14	–	–	–	29/61	160	235	1448*
P/*Brueelia kluzi* Balát, 1955**	0/27	–	–	–	0/14	–	–	–	2^1^/61	11	11	17
*Serinus canaria* (Linnaeus, 1758)	0/19	–	–	–	0/11	–	–	–	–	–	–	–
Family Muscicapidae												
*Erithacus rubecula rubecula* (Linnaeus, 1758)												
C/*Dasypsyllus gallinulae* (Dale, 1878)	1/10	0	1	–	0/6	–	–	–	0/3	–	–	–
H/*Ornithoica turdi* (Olivier in Latreille, 1811)***	0/10	–	–	–	2/6	1	1	–	0/3	–	–	–
P/*Guimaraesiella tristis* (Giebel, 1874)	2/10	23	38	12	0/6	–	–	–	0/3	–	–	–
R/*Ricinus rubeculae* (Schrank, 1776)	1/10	0	5	4	0/6	–	–	–	0/3	–	–	–
Family Passeridae												
*Passer domesticus domesticus* (Linnaeus, 1758)												
C/*Ceratophyllus* (*Ceratophyllus*) *gallinae* (Schrank, 1803)	2/16	0	2	–	1/1	0	1	–	0/2	–	–	–
H/*Ornithomya avicularia* (Linnaeus, 1758)	1/16	0	1	–	0/1	–	–	–	0/2	–	–	–
Family Regulidae												
*Regulus regulus azoricus* Seebohm, 1883												
P/*Philopterus gustafssoni* Najer et al., 2020	5/5	12	7	38	–	–	–	–	–	–	–	–
*Regulus regulus sanctaemariae* Vaurie, 1954												
P/*Philopterus gustafssoni* Najer et al., 2020	–	–	–	–	1/10	0	1	0	–	–	–	–
Family Sylviidae												
*Sylvia atricapilla gularis* Alexander, 1898												
C/*Dasypsyllus gallinulae* (Dale, 1878)**	4/17	7	0	–	0/24	–	–	–	0/5	–	–	–
M/*Myrsidea sylviae* Sychra & Literak, 2008	11^2^/17	13	7	21	11^2^/24	6	4	13	2^2^/5	3	1	0
P/*Guimaraesiella tovornikae* (Balát, 1981)	12^2^/17	39	63	65	5^2^/24	12	10	16	3^2^/5	5	3	6
Family Turdidae												
*Turdus merula azorensis* Hartert, 1905												
C/*Ceratophyllus* (*Ceratophyllus*) sp.***	0/11	–	–	–	1/18	1	0	–	0/4	–	–	–
C/*Dasypsyllus gallinulae* (Dale, 1878)**	2/11	1	1	–	0/18	–	–	–	0/4	–	–	–
H/*Ornithomya avicularia* (Linnaeus, 1758)**	1/11	0	1	–	0/18	–	–	–	0/4	–	–	–
H/*Ornithoica turdi* (Olivier in Latreille, 1811)**	1/11	0	1	–	9/18	3	15	3^FM^	4/4	0	5	1^FM^
M/*Menacanthus eurysternus* (Burmeister, 1838)**	2^3^/11	0	1	1	1/18	0	1	0	3/4	7	7	11
P/*Guimaraesiella amsel* (Eichler, 1951)**	8/11	29	89	77	1/18	0	1	0	1^1^/4	2	2	4
P/*Philopterus turdi* (Denny, 1842)**	1^1^/11	0	1	0	0/18	–	–	–	0/4	–	–	–
P/*Sturnidoecus* sp.***	0/11	–	–	–	4^4^/18	3	4	0	0/4	–	–	–
P/*Turdinirmus merulensis* (Denny, 1842)**	0/11	–	–	–	8^4^/18	4	21	2	0/4	–	–	–
Siphonaptera total	12/107	8	7	–	2/84	1	1	–	2/75	1	1	–
Hippoboscidae total	3/107	0	3	–	11/84	4	16	3^FM^	15/75	3	16	1^FM^
Phthiraptera total	32/107	116	211	226	25/84	25	41	31	37/75	188	259	1486*

1 Previous chewing lice species was found on the same host/s;

2 Co-occurrence of both species of chewing lice was found on nine birds in São Miguel, three birds in Santa Maria, and two birds in Graciosa.

3 *Guimaraesiella amsel* was found on the same hosts;

4 Co-occurrence of both species of chewing lice was found on three birds;

* 104 males, 135 females and 1078 nymphs were collected and at least 60 other specimens were observed on one host;

** New parasite-host record for examined subspecies of host;

*** New parasite-host record for examined species of host.

A total of 16 (6.0%) birds from five species were parasitized by 19 fleas of three species: *Ceratophyllus* (*Ceratophyllus*) *gallinae* (Schrank, 1803), *Ceratophyllus* (*Ceratophyllus*) sp. ex *Turdus merula*, and *Dasypsyllus gallinulae* (Dale, 1878). The total mean intensity was 1.2, 1–3 fleas were collected from each host, and no host individual was parasitized by more than one species of flea. While fleas of the genus *Ceratophyllus* were found on one host species, *D. gallinulae* was found on four host species (Table 1).

A total of 29 (10.9%) birds representing four species were parasitized by 43 louse-flies of two species: *Ornithomya avicularia* (Linnaeus, 1758)*, Ornithoica turdii* (Olivier in Latreille, 1811). No host individual was parasitized by more than one species of fly. Both species were more abundant in September; only three individuals (one *O. turdi* and two *O. avicularia*) were collected in April. Each species was collected from three different host species (Table 1).

A total of 91 (34.2%) birds representing five species were parasitized by 11 species of chewing lice (Table 1). A total of 12 louse-host associations were found, which represents only about 1/3 of the known louse-host associations (*n* = 38) for these eight examined passerine bird species outside the Azores (Supplementary Table S1). Most birds (80.2%, *n* = 91) showed only very light to light infestations (1–20 lice/host; Supplementary Table S2). Extremely heavy infestations were found on two *F. coelebs* from Graciosa, parasitized by 175 and more than 1350 individuals of *Menacanthus eurysternus* (Burmeister, 1838), respectively (see Discussion).

Most birds (74.7%, *n* = 91) were parasitized with only one species of chewing louse, but co-occurrence of two species of lice was recorded on 23 birds. In 19 cases, co-occurrence of one ischnoceran and one amblyceran louse species was recorded, and in the remaining four cases, two species of ischnoceran lice were recorded (Table 1). The most frequent co-occurrence of lice infesting *S. atricapilla* included *Guimaraesiella tovornikae* and *Myrsidea sylviae*. We found no significant difference in prevalence, mean intensity or mean abundance of these lice on hosts harbouring both species or on those harbouring only *Guimaraesiella* or only *Myrsidea*. So it seems there is probably no interaction between these two species of lice, at least at levels of infestation observed in this study. Co-occurrence of two species of lice was also found on *F. coelebs* and *T. merula* (see notes and data in Table 1). All species of chewing lice were found on only one host species with one exception, *M. eurysternus*, which was recorded on two species of birds. Dominance among the eight genera of lice is ranked as follows: *Menacanthus* (73.1%), *Guimaraesiella* (19.0%), *Myrsidea* (2.6%), *Philopterus* (2.3%), *Brueelia* (1.5%), *Turdinirmus* (1.0%), *Ricinus* (0.3%) and *Sturnidoecus* (0.3%, *n* = 2617). Dominance is strongly affected by aforementioned extreme infestation of 1350 individuals of *M. eurysternus*.

There were no significant differences in infestation parameters for total lice on the three species of birds with conspicuous sexual dimorphism and for which samples sizes were greatest: *F. coelebs, S. atricapilla* and *T. merula*.

A total of 114 (42.9%) birds from six species were parasitized by 1–4 species of insect ectoparasites. Most birds (81.6%, *n* = 114) were parasitized with only one species of ectoparasite. Thirteen birds were parasitized by two species (chewing louse and louse-fly or chewing louse and flea), seven birds by three species (two chewing lice and louse-fly or two chewing lice and flea) and on one *T. merula* was infested by four species (*G. amsel*, *M. eurysternus*, *O. turdi* and *D. gallinulae*). In total, the highest diversity of insect ectoparasites was found on *T. merula* with nine recorded species: two species of fleas, two species of louse-flies and five species of chewing lice were found on this bird species (Table 1).

We recorded one case of phoresy by one female of *Guimaraesiella amsel* on *Ornithoica turdi* collected on 15 April from *T. merula* (No. AZ32) on São Miguel. This same host individual was also infested by 13 additional specimens of *G. amsel*. *Turdus merula* is the only host species of this species of chewing louse, and birds No. AZ30 and AZ32, both examined on 15 April and harbouring *G. amsel*, were the first blackbirds examined by us in São Miguel. We found one female and one male of *G. amsel* on *F. coelebs* (No. AZ24) and *R. r. azoricus* (No. AZ29) at the same locality on 14 and 15 April, respectively. Similarly, we found one male and one female of *G. amsel* on *S. atricapilla* (No. AZ56) examined on 16 April at 13:50. The closest blackbird with *G. amsel* (No. AZ47) was examined on the same day at 10:05. The case of *G. tovornikae*, a specific parasite of *S. atricapilla*, infesting *T. merula* (No. AZ47) is also unlikely to be a case of contamination in the field. This blackbird was the first bird examined for lice on that day of collection and it was taken at a different location from previous birds (AZ01-AZ43).

### Redescriptions

PHTHIRAPTERA Haeckel, 1896

Amblycera Kellogg, 1896

Menoponidae Mjöberg, 1910

*Myrsidea* Waterston, 1915

Myrsidea sylviae Sychra & Literak, 2008

**Figure 1:**
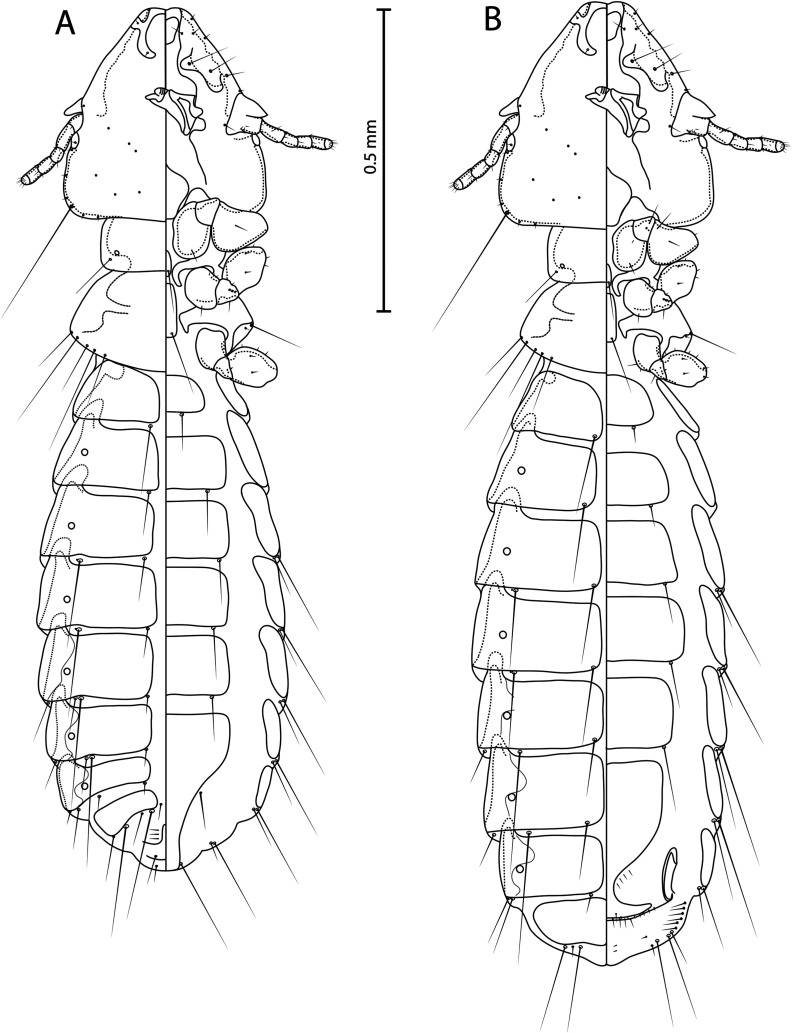
*Guimaraesiella tovornikae* ex *Sylvia atricapilla*. (A) Male dorso-ventral view; (B) Female dorso-ventral view.

**Figure 2:**
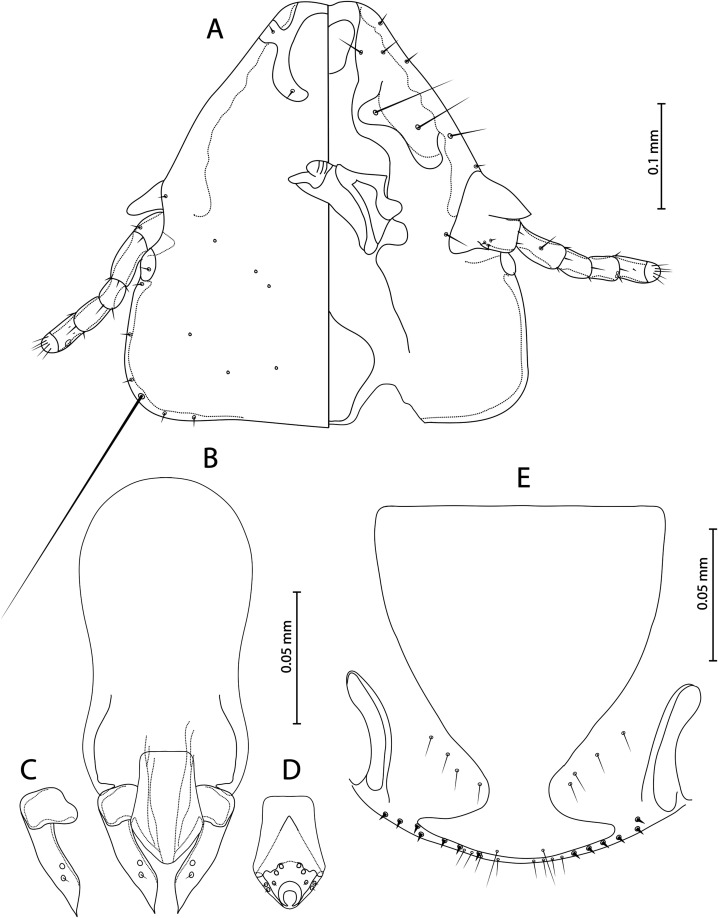
*Guimaraesiella tovornikae* ex *Sylvia atricapilla*. (A) Male head, dorso-ventral view; (B) Male genitalia, dorsal view; (C) Male paramere, dorsal view; (D) Male mesosome, ventral view; (E) Female submarginal plate and vulval margin, ventral view.

**Figure 3:**
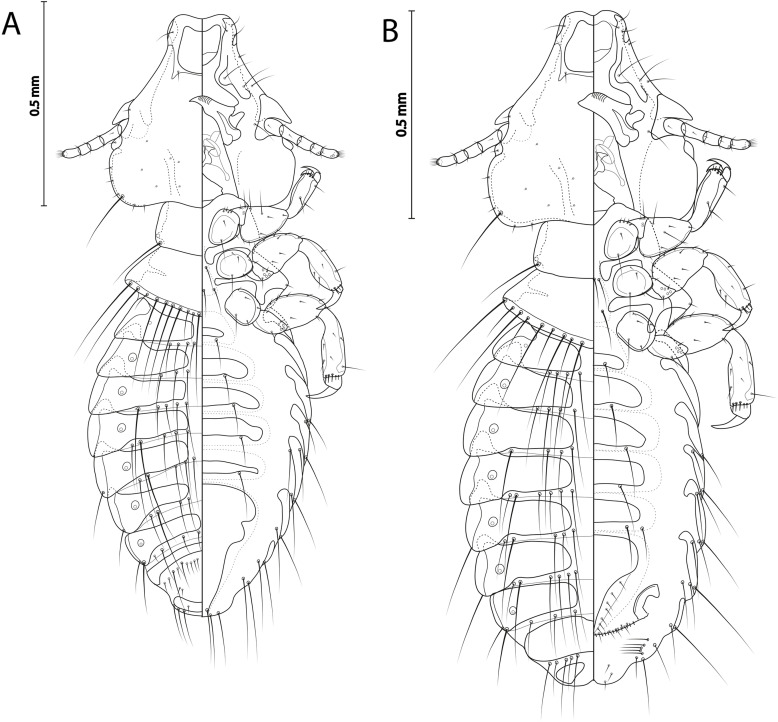
*Sturnidoecus* ex *Turdus merula.* (A) Male dorso-ventral view; (B) Female dorso-ventral view.

*Myrsidea sylviae* Sychra & Literak, 2008: 241: Figures 1–3.

**Type host**: *Sylvia atricapilla atricapilla* (Linnaeus, 1758) – Eurasian blackcap (Sylviidae).

**Type locality**: Čerťák, Czech Republic

**Remarks.** *Myrsidea sylviae* was described by Sychra and Literak [71] on the basis of one male and one female from *S. atricapilla* in the Czech Republic. On the basis of specimens from the Azores, we update the knowledge on intraspecific morphological variability of this species of louse. The original data concerning type specimens described by Sychra and Literak [71] are in parentheses.

**Female** (*n* = 5). Hypopharyngeal sclerites fully developed. Length of *dhs 10*, 0.06–0.07 (0.06); *dhs 11*, 0.09–0.10 (0.09); ratio *dhs 10/11*, 0.63–0.72 (0.67). Labial setae 5, 0.04 long (broken on holotype), latero-ventral fringe with 9–11 setae. Gula with 4–5 (4) setae on each side. Pronotum with 3 setae on posterior margin and 3 short spiniform setae at each lateral corner. Prosternal plate with rounded anterior margin. First tibia with 3 outer ventro-lateral and 4 dorso-lateral setae. Mesonotum divided. Metanotum not enlarged, with 5–7 (5) marginal setae (the most posterolateral setae are not counted); metasternal plate with 6 setae; metapleurites with 3 short strong spiniform setae. Femur III with 13–15 (15) setae in ventral setal brush. Tergites not enlarged with small medioposterior convexity on segments II–IV. Abdominal segments with well-defined median gap in each row of tergal setae. Tergal setae (postspiracular setae and on tergites II–VIII also short associated setae are not included): I, 8–10 (10); II, 11; III, 12–14 (14); IV, 10–11 (11); V, 9–10 (10); VI, 8–10 (10); VII, 5–6 (6); VIII, 4. Postspiracular setae very long on II, IV and VIII (0.39–0.47); long on I and VII (0.31–0.35); and short on III, V and VI (0.11–0.20). Inner posterior seta of last tergum as long as anal fringe setae with length 0.08–0.09 (0.08); length of short lateral marginal seta of last segment, 0.04–0.06 (0.04). Pleural setae: I, 5–6; II, 7; III, 8; IV, 6–7; V, 4–6; VI, 4–5; VII, 3–4; VIII, 3. Pleurites with only short spine-like setae, without anterior setae. Pleurite VIII with inner setae (0.04) as long as outer (0.03–0.04). Anterior margin of sternal plate II with a medial notch. Sternal setae: I, 0; II, 4 in each aster, aster setae length: *s1*, 0.03–0.04; *s2*, 0.05–0.06; *s3*, 0.06; *s4*, 0.09–0.10; with 14–16 (16) marginal setae between asters, 4–6 (6) medioanterior; III, 21–28 (28); IV, 27–37 (37); V, 31–36 (36); VI, 20–27 (27); VII, 10–11 (11); VIII–IX, 8–9 (8); and 5–7 (5) setae on deeply serrated vulval margin; sternites III–VII without medioanterior setae. Anal fringe formed by 35–36 dorsal and 35–40 ventral setae. Dimensions: HW = 0.40–0.41 (0.40); POW = 0.32; HL = 0.31–0.32 (0.32); PW = 0.25; MW = 0.37–0.41 (0.41); AW = 0.54–0.59 (0.59); ANW = 0.19–0.20 (0.20); TL = 1.43–1.53 (1.53).

**Male** (*n* = 5). Similar to female except as follows. Length of *dhs 10*, 0.05–0.07 (0.06); *dhs 11*, 0.09–0.10 (0.10); ratio *dhs 10/11*, 0.51–0.70 (0.60). Labial setae 5 0.03–0.04 (0.04) long. Gula with 4–7 (5) setae on each side. Metanotum with 4–6 (4) marginal setae (the most posterolateral setae are not counted). Femur III with 13–14 (14) setae in ventral setal brush. Abdominal segments with well-defined median gap in each row of tergal setae. Tergal setae (postspiracular setae and on tergites II–VIII also short associated setae are not included): I, 6–9 (8); II, 7–9 (9); III, 9–10 (10); IV, 8–10 (9); V, 9–10 (9); VI, 6–10 (10); VII, 5–8 (8); VIII, 4–6 (6). Postspiracular setae very long on II, IV and VIII (0.39–0.44); long on I and VII (0.18–0.31); and short on III, V and VI (0.07–0.13). Length of inner posterior seta of last tergum, 0.06–0.08 (0.08); short lateral marginal seta of last segment, 0.02. Pleural setae: I, 3–4; II, 5–6; III, 6–7; IV, 6; V, 5–6; VI, 4–5; VII, 3–4; VIII, 3. Pleurite VIII with inner setae (0.04) as long as outer (0.03–0.04). Sternal setae: I, 0; II, 4 in each aster, aster setae length: *s1*, 0.03–0.04; *s2*, 0.04–0.05; *s3*, 0.05–0.07; *s4*, 0.07–0.10; with 12–13 (12) marginal setae between asters, 4–7 (7) medioanterior; III, 15–20 (18); IV, 24–28 (27); V, 25–27 (26); VI, 20–21 (21); VII, 10–11 (11); VIII, 6–8 (8); remainder of plate, 5–7 (7); and with 3–5 (3) setae posteriorly; sternites III–VII without medioanterior setae. With 8–9 (8) internal anal setae. Dimensions: HW = 0.37–0.38 (0.38); POW = 0.30; HL = 0.28–0.29 (0.28); PW = 0.23–0.25 (0.25); MW = 0.30–0.34 (0.34); AW = 0.44–0.46 (0.46); GW = 0.10–0.11 (0.11); GL = 0.36–0.40 (0.40); ParL = 0.06–0.09 (0.07); GSL = 0.08–0.10 (0.09); TL = 1.17–1.22 (1.22).

**Examined material. Holotype** ♀ ex *Sylvia atricapilla atricapilla*, Čerťák, Czech Republic, 31 Aug. 2005, O.Sychra-CZ84 (MMBC). **Paratype** ♂, same host and locality, 23 Apr. 2007, O.Sychra-CZ85 (MMBC).

Other material. Non-types ex *Sylvia atricapilla gularis*: 2♀, 2♂, São Miguel, Azores, Portugal, 16 Apr. 2013; 2♀, 2♂, Santa Maria, Azores, Portugal, 18 Sep. 2013; 1♀, 1♂, Graciosa, Azores, Portugal, 25 Sep. 2013.

Ischnocera Kellogg, 1896

Philopteridae Burmeister, 1838

*Brueelia*-complex

*Guimaraesiella* Eichler, 1949

#### *Guimaraesiella tovornikae* (Balát, 1981)

Figures 1–2, 5A, 5B

**Figure 4:**
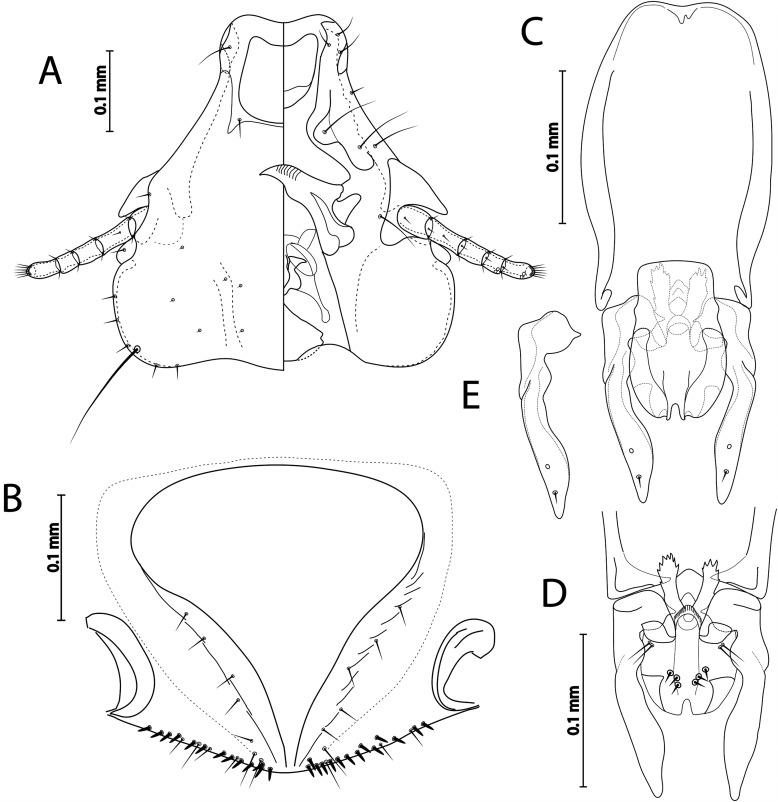
*Sturnidoecus* ex *Turdus merula.* (A) Male head dorso-ventral view; (B) Female subgenital plate and vulval margin, ventral view; (C) Male genitalia dorsal view; (D) Male genitalia, ventral view; (E) Male paramere, dorsal view.

*Allonirmus tovornikae* Balát, 1981: 281: Figures 4, 14, plate IV, Figures 3, 6.

*Nigronirmus atricapillae* Soler-Cruz et al., 1984: 147: Figures 3 and 4.

*Brueelia neoatricapillae* Price, et al. [in Price et al.], 2003.

*Guimaraesiella tovornikae* (Balát, 1981); Gustafsson and Bush 2017: 222.

**Type host**: *Sylvia atricapilla atricapilla* (Linnaeus, 1758) – Eurasian blackcap (Sylviidae).

**Type locality**: Antošovice, Czech Republic

**Remarks.** *Guimaraesiella tovornikae* was described as *Allobrueelia tovornikae* by Balát [4] on the basis of few specimens found on *S. atricapilla* in the Czech Republic. Gustafsson & Bush [24] considered *Allobrueelia* to be a synonym of *Guimaraesiella* Eichler, 1949. Recently, all *Brueelia sensu lato* described by Balát were redescribed [26] with one exception – *G. tovornikae* – because type specimens of this species were not available to us at that time. Here we redescribe *G. tovornikae* based on Balát’s type series and also on material from *S. atricapilla* from the Azores to update Balát’s description and to increase the knowledge of intraspecific variability of this species. The measurements of holotype male and paratype female are in parentheses.

We have not examined the type material of *Nigronirmus atricapillae* Soler-Cruz et al. [70]. The original illustrations of *N. atricapillae* are largely consistent with the morphology of *A. tovornikae*. However, several setae are misplaced in the original illustrations of *N. atricapillae*, including several ventral head setae that have been illustrated dorsally, and vice versa, and some abdominal setae that have been duplicated and illustrated both dorsally and ventrally. Moreover, the female subgenital plate of *N. atricapillae* is incorrectly illustrated as similar to other sternal plates in their Figure 3, but not in their Figure 4. We therefore accept the synonymy of this species with *A. tovornikae* proposed by Gustafsson and Bush [24].

*Brueelia neoatricapillae* was proposed as a replacement name for *Brueelia atricapillae*, under the assumption that this name was preceded by *Brueelia atricapilla* Cicchino, 1983 (now in *Guimaraesiella*). However, the *– a/-ae* difference is not listed among the exceptions to the “one-letter difference” rule in the Code (International Commission on Zoological Nomenclature, 1999; Articles 57.6 and 58). These two names are therefore not homonyms, and *Brueelia neoatricapillae* is an unnecessary replacement name, which automatically becomes a synonym of *N. atricapillae*.

**Figure 5:**
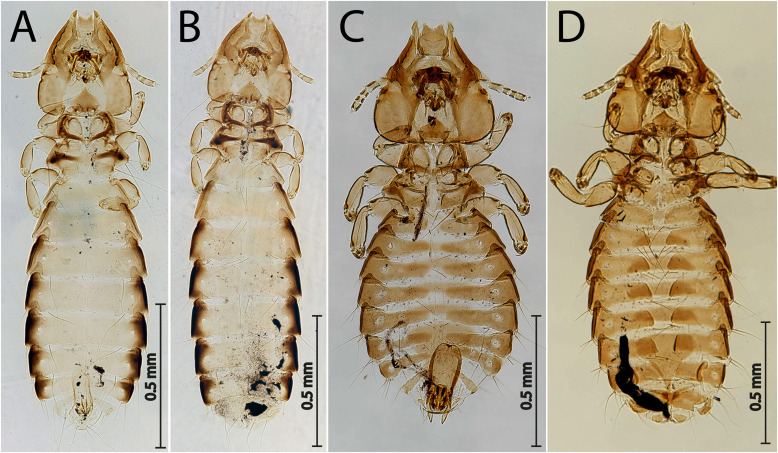
Habitus: *Guimaraesiella tovornikae* ex *Sylvia atricapilla*. (A) Holotype male; (B) Paratype female. *Sturnidoecus* ex *Turdus merula;* (C) Male; (D) Female.

**Both sexes**. Basic characteristic, i.e. head structure, thoracic and abdominal segments, chaetotaxy, and male genitalia as described for the genus in Gustafsson and Bush [24]. Light yellow to brown body colour. Head broad, pentagonal flat-shaped (Fig. 2A), frons concave. Marginal carina interrupted submedianly. Dorsal preantennal suture reaching *ads* and lateral head margin, not encircling dorsal anterior plate, ventral anterior plate present. Triangular coni reaching distal margin of scapes, antennae sexually monomorphic. Preantennal nodi with bulge in distal end, preocular nodi wider than postocular, both much wider than temporal marginal carina. Temporal marginal carina thin, less prominent. Gular plate pentagonal. Head chaetotaxy as in Figure 2A. Prothorax rectangular, posterior margin of prothorax flat. Median ends of proepimera hook-shaped. Pterothorax rounded-pentagonal. Median ends of metepisterna hammer-shaped. Mesosternum and metasternum little visible, translucent. Sternal plates roughly rectangular, not reaching pleurites. Re-entrant heads of pleurites III–VII translucent. Increasing pigmentation of pleurites from segment IV to segment VII (Figs. 5A and 5B). Thorax and abdomen chaetotaxy as in Figures 1 and 2; *ss* present on segments II–VIII; *sts* present on segments II–VI; *ps* present on segments IV–VII.

**Male** (*n* = 23). Abdominal chaetotaxy as in Figure 1A; *psps* present on segments IV–VIII; *aps* present on segments VI–VII, in three individuals *aps* present also on one side of segment V. Subgenital plate with concave lateral margins in distal half (Fig. 2B). Mesosome pentagonal-shaped with flat proximal and rounded distal margin, ventral sclerite pointed, gonopore open distally, mesosomal lobes thin, rugose nodi absent (Fig. 2D). Mesosomal chaetotaxy typical for genus (Fig. 2D). Parameral heads roughly quadratic, parameral blades short and pointed distally with *pst 1*–*2* (Fig. 2C). Dimensions: HW = 0.30–0.33 (0.30); HL = 0.30–0.34 (0.34); PW = 0.17–0.19 (0.19); PTW = 0.25–0.28 (0.26); AW = 0.36–0.42 (0.39); TL = 1.20–1.41 (1.32).

**Female** (*n* = 16). Abdominal chaetotaxy as in Figure 1B; *psps* present on segments IV–VII. Subgenital plate roughly triangular, approaching vulval margin, not cross-piece (Fig. 2E). Vulval margin with 3–5 slender *vms* and 4–10 thorn-like *vss* on each side of subgenital plate; lateral margins with 2–6 *vos* on each side; distal 1 *vos* median to *vss*. Dimensions: HW = 0.33–0.36 (0.33); HL = 0.34–0.37 (0.37); PW = 0.20–0.22 (0.21); PTW = 0.29–0.31 (0.29); AW = 0.43–0.47 (0.44); TL = 1.54–1.70 (1.62).

**Examined material. Holotype** ♂ ex *Sylvia atricapilla atricapilla*, Antošovice, Czech Republic, 2 Jul. 1977, FB 1383 (MMBC). **Paratypes** 2♂, 1♀, same collection data as holotype, FB 1382a–b (MMBC).

Other material. Non-types ex *Sylvia atricapilla atricapilla*: 1♀, Čerťák, Czech Republic, 31 Aug. 2005; 3♂, 1♀, Stozice, Ljubljana, Slovenia, 15 May 1978, D. Sere, 13264–7 (PMSL); 2♂, Stozice, Ljubljana, Slovenia, 19 Sep. 1978, D. Sere, 13483–4 (PMSL); 2♂, 8♀, Ljubljana, Slovenia, 15 Aug. 1960, S. Brelih, 2975–6, 2978–85 (PMSL); 1♀, Tomacevo, Ljubljana, Slovenia, 22 Apr. 1974, S. Brelih, 11460 (PMSL); 1♀, Tomacevo, Ljubljana, Slovenia, 10 Apr. 1975, D. Sere, 12835 (PMSL); 6♂, 6♀, Metkovic, Croatia, 23 Apr. 1963, A. Lesinger, 8401, 8403–8, 8410–4 (PMSL); ex *Sylvia atricapilla gularis*: 11♀, 16♂, São Miguel, Azores, Portugal, 16 Apr. 2013; 2♀, 4♂, Santa Maria, Azores, Portugal, 18–19 Sep. 2013; 1♀, 2♂, Graciosa, Azores, Portugal, 25 Sep. 2013.

Note: Sychra et al. [72] reported one female of *Brueelia tovornikae* and one female of *Brueelia neoatricapillae* Price, Hellenthal, Palma, 2003 on *S. atricapilla* from the Czech Republic. Since Gustafsson & Bush [24] synonymized *B. neoatricapillae* with *B. tovornikae* and moved this species to the genus *Guimaraesiella*, we re-examined aforementioned females and found that one represents *G. tovornikae* (it is included in examined material). However, the specimen reported as *B. neoatricapillae*, was incorrectly identified, and represents *G. amsel*, a parasite of *T. merula*. We suspect this female is a contaminant as a result of the handling of ringed birds.

#### *Sturnidoecus* sp.

Figures 3–4, 5C, 5D.

**Remarks**. No species of *Sturnidoecus* is known from *T. merula* [24], and our record thus represents a new host record. The specimens belong to the *S. simplex* species group *sensu* Gustafsson & Bush [24], which is commonly found on thrushes in the genus *Turdus*. However, the species of this group are poorly known, and most cannot presently be identified to species level; the group is in need of revision. Moreover, preliminary investigations of the morphological variation within this group suggest that differences among species are minor, and that some proposed names may be better considered synonyms (DR Gustafsson, unpublished data). For this reason, we cannot presently determine whether these specimens represent a new species of *Sturnidoecus*, or a known species occurring on a new host species. Rather than describing the species as new, we here present morphological data for the specimens we have collected, in anticipation of a revision of the *Sturnidoecus simplex* species group.

**Both sexes**: Basic characteristic, i.e. head shape, structure, thoracic and abdominal segments, chaetotaxy, and male genitalia as described for the genus and *simplex* species group in Gustafsson & Bush [24]. Head as in Figure 4A. Head bulb-shaped, frons concave, dorsal anterior plate with concave anterior margin and flat posterior margin, dorsal preantennal suture completely encircling dorsal anterior plate. Dorsal preantennal suture extends only slightly towards blunt preantennal nodi. Ventral anterior plate present. Coni long, pointed, reaching well beyond distal margin of scapes, antennae sexually monomorphic. Preocular nodi rectangular, postocular nodi rounded, both wider than temporal marginal carina. Temporal marginal carina thin. Gular plate as in Figure 4A. Temporal carina well visible. Prothorax rectangular, median ends of proepimera hammer-shaped. Posterior margins of pterothorax convergent to median point. Median ends of metepisterna slender. Meso- and metasternum translucent. Tergopleurites rectangular. Sternal plates large, present on segments II–VI. Re-entrant heads on pleurites III–VII. Translucent areas around spiracles.

**Male** (*n* = 3). As in Figures 3A and 5C. Pterothorax with continuous row of 20–21 marginal mesometanotal setae. Abdominal chaetotaxy as in Figure 3A with number of setae on each side as in Table 2. Male genitalia as in species group description in Gustafsson & Bush [24] and Figures 4C–4E. Subgenital plate reach posterior margin of abdomen. Dimensions: HW = 0.46–0.48; HL = 0.46–0.47; PW = 0.26–0.27; PTW = 0.40–0.43; AW = 0.54–0.66; TL = 1.39–1.50.

**Table 2:** Chaetotaxy of one side of abdominal segments II–IX of male and female of *Sturnidoecus* from *Turdus merula* collected in the Azores, 2013. Trichoid setae of segment VIII are present in all specimens, and are not listed. Abbreviations: *aps = accessory post-spiracular seta; psps = principal post-spiracular seta; ps = paratergal seta; ss = sutural seta; sts = sternal seta; tps = tergal posterior seta.*

	*aps*		*psps*		*tps*		*ss*		*sts*		*ps*	
	Male	Female	Male	Female	Male	Female	Male	Female	Male	Female	Male	Female
II					2–3	3	1	1	1	1		
III					2–4	3	1	1	1	1		
IV			1	1	2–3	2–3	1	1	1	1	2	2
V	(0–) 1*	1	1	1	2–3	2–3	1	1	1	1	2–3	3
VI	1	1	1	1	1–3	2–3	1	1	1	1(–2)*	3	4
VII	1	(0–)1*	1	1	2–3	2–3	1	1			3	4
VIII	0–1**		1	1	1–2	1–2	1	1			4	4
IX					8–10	5						

* One *aps* is not present or one more *sts* is present on one side of one specimen;

** *aps* are not present in one examined male.

**Female** (*n* = 2). As in Figures 3B and 5D. Pterothorax with continuous row of 18–23 marginal mesometanotal setae. Abdominal chaetotaxy as in Figure 4A with number of setae on each side as in Table 2. Subgenital plate as in Figure 4B, cross-piece not present. Vulval margin gently rounded, with 4–6 slender *vms* on each side, and 10–13 thorn-like *vss* on each side; and 7 slender *vos* on each side. Dimensions: HW = 0.50–0.51; HL = 0.50; PW = 0.29–0.30; PTW = 0.44–0.45; AW = 0.60–0.62; TL = 1.63–1.72.

**Examined material.** 2♀, 3♂ ex *Turdus merula azorensis*, Santa Maria, Azores, 20. Sep. 2013.

## Discussion

This is the first comprehensive study focused on insect ectoparasites on wild passerines from the Archipelago of the Azores. We focused on three groups of parasites: fleas, louse-flies and chewing lice.

### Records of fleas

The fleas *Ceratophyllus* (*Ceratophyllus*) *gallinae* and *Dasypsyllus gallinulae* have been reported from the Azores, but only from São Miguel [8]. Our records represent the first finding of these species on Santa Maria and Graciosa. All parasite-host associations we recorded are known for continental populations of the same hosts [66]. The only exception is *Ceratophyllus* (*Ceratophyllus*) sp. on *T. merula*, which represents a new parasite-host association. Eight different species of *Ceratophyllus* have been found on *T. merula* in different parts of Europe: *C.* (*Ceratophyllus*) *fringillae* (Walker, 1856), *C.* (*Ceratophyllus*) *gallinae* (Schrank, 1803), *C.* (*Ceratophyllus*) *pullatus* Jordan & Rothschild, 1920, *C.* (*Ceratophyllus*) *tribulis* Jordan, 1926, *C.* (*Emmareus*) *borealis* Rothschild, 1907, *C.* (*Emmareus*) *columbae* (Gervais, 1844), *C.* (*Emmareus*) *garei* Rothschild, 1902, and *C.* (*Monopsyllus*) *sciurorum sciurorum* (Schrank, 1803) [20, 54]. The specimen recorded on *T. merula* belongs to subgenus *Ceratophyllus* but it shows a combination of features which do not allow its identification to species level. It is possible the specimen is an anomaly or it represents an undescribed species (D Cyprich and TD Galloway, pers. comm.). Additional material is necessary to resolve this interesting record.

### Records of louse-flies

Both species of louse-flies we recorded (*Ornithoica turdi* and *Ornithomya avicularia*) represent new records for the Azores, as well as new westernmost distribution records of each species. *Ornithomya avicularia* is widely distributed in the Palearctic region, but with limited records in the Ethiopian region [74], and has been recorded from all three Azorean hosts (*F. coelebs*, *P. domesticus*, and *T. merula*) on the mainland [15]. In contrast, *O. turdi* is widely distributed in the Ethiopian region and southern Palearctic, with a recent increase in records from Central Europe [23], where it has been recorded from two of the three host species recorded here (*F. coelebs*, and *T. merula*) [15]. The third host we recorded for *O. turdi*, *E. rubecula*, represents a new host-parasite association for this louse-fly species [23, 32, 36, 37]. *Ornithoica turdi* was recorded from all three islands in this study, whereas *O. avicularia* was found only on São Miguel and Graciosa. Louse-flies of both species were more abundant in September than in April. It is in accordance with their life cycles as their imagoes occur mainly from August to October [15].

### Records of lice

Chewing lice were the most abundant insect ectoparasites recorded on wild passerines in the present study. A total of 11 species of chewing lice belonging to eight genera were found. Each of these genera are near globally distributed on passerines, being absent only from Antarctica [24, 44, 48, 55, 56, 58, O Sychra unpublished data].

*Ricinus rubeculae* is an euryxenous host generalist louse species known from 14 species of passerine birds, including *Erithacus rubecula* [57, 58], which is the only Azorean host found to date. On the mainland, *R. rubeculae* is widely distributed mainly in warmer areas of Palearctic, and Oriental Regions [58], and our record represents a new westernmost distributional limit of this species.

*Menacanthus eurysternus* is a euryxenous generalist with cosmopolitan distribution [39, 55]. Our record confirms its occurrence also in the Archipelago of the Azores. An interesting case of a very high infestation (over 1350 individuals) by this haematophagous species was recorded on a male of *F. coelebs* in September, i.e. post-breeding period, on Graciosa. To our knowledge, this is the highest infestation ever reported for this species [14]. Such a high infestation would normally be expected on birds in poor health or with reduced ability to preen or scratch [57]. However, the examined male was apparently in good condition, coloured as other examined males without visible injury, deformation of the bill or legs. We can only speculate that it may have been weakened by some disease or an internal parasite, but this was not tested by us.

*Myrsidea sylviae* was originally described by Sychra and Literak [71] on the basis of one male and one female in *S. atricapilla* in the Czech Republic. Moreover, both specimens were found on different host individuals at different times of the year (female on one of 75 examined birds in August 2005 and male on one of 110 examined birds in April 2007). Despite the fact that this species differed from other *Myrsidea* species in Europe, collection of only two individuals made its status and association with *S. atricapilla* questionable. However, more recently Literak et al. [34] found a large population of *M. sylviae* on *S. atricapilla* in the Azores. It confirms that this bird is really a natural host of this louse species. Literak et al. [34] considered that the dissimilarity in population sizes and prevalence of *M. sylviae* between the Azores and mainland Europe may be influenced by the migratory behaviour of its hosts – resident populations of *S. atricapilla* on the Azores vs. migratory ones on the mainland. Other ecological factors certainly may play a role, e.g. lice in the genus *Myrsidea* have been suggested to be distributionally limited by ambient humidity [11], but more data are needed to test whether this may explain these differences between the Azores and Europe.

All species from the *Brueelia-*complex recorded in the Azores, i.e. *B. kluzi*, *G. amsel*, *G. tovornikae*, *G. tristis* and *T. merulensis* are strictly host-specific lice known from their type hosts from different areas of continental Europe [24, 26, 73]. Our records represent a new westernmost distributional limit of all these species. Moreover, the host subspecies recorded here for *B. kluzi, G. amsel,* and *T. merulensis* represent new host-louse associations.

*Philopterus gustafssoni*, formerly *Philopterus reguli* [46], is a parasite of *Regulus regulus* (Linnaeus, 1758) and *Regulus ignicapilla* (Temminck, 1820), frequently reported from continental Europe [46]. Recently, Najer et al. [46] confirmed that *Philopterus* lice collected from endemic subspecies of goldcrests – *R. r. azoricus* from São Miguel and *R. r. sanctaemariae* from Santa Maria – are conspecific with *P. gustafssoni* (*P. reguli*) from the mainland. No subspecies of goldcrest occurs on Graciosa [59].

The specimens of *Sturnidoecus* from *T. merula* represent a new parasite-host association [24]. *Turdus merula* is a common bird in Europe and its chewing louse fauna, which comprises seven species, is well known in this continent [7, 10, 24, 57, Supplementary Table S1]. The specimens we collected are part of the *S. simplex* species group *sensu* Gustafsson & Bush [24]. This species group contains 14 species, of which 11 parasitize thrushes in the genus *Turdus* from the Nearctic and Neotropical regions, and 2 species are found on *Onychognathus* starlings in Africa. The remaining species, *S. melodicus* (Eichler, 1951), was described based on one female collected from a European *Turdus philomelos* Brehm, 1831. Our records thus constitute the second record of a louse in the *S. simplex* species group from the Palearctic.

The paucity of records of this species group from the Old World, compared to the wealth of species and records from the New World, suggests that our records may originate from some New World host. Our specimens from the Azores are very similar to those of *Sturnidoecus simplex* (Kellogg, 1896), a widespread parasite of *Turdus migratorius* Linnaeus, 1766 [24]. This bird species is known as an occasional migrant on the Azores [1, 5]. One possible scenario could be a host-switch of the *Sturnidoecus* lice from *T. migratorius* to *T. merula*, which would explain the absence of *Sturnidoecus* on *T. merula* on the European mainland. On the other hand, up to now *T. migratorius* has been recorded only on Corvo, one of the westernmost islands of the archipelago, while *Sturnidoecus* were found on *T. merula* on the easternmost island of Santa Maria.

Alternatively, our records may represent some known or unknown Old World species of *Sturnidoecus*. Neither of the two sturnid hosts of *S. simplex* species group lice occur anywhere near the Azores [9]; none of the *Onychognathus* starlings that occur in Western Africa are known to be parasitized by any species of *Sturnidoecus*, but records of the *Brueelia*-complex of lice from Africa are very sparse [24, 27], and these may simply have been overlooked. The only other Old World record of this species group is *S. melodicus* from Europe, which is known from one specimen collected from *Turdus philomelos* from Germany. If our specimens are close to or conspecific with *S. melodicus*, this may represent a relict population of a species of louse on the Azores, one which has all but disappeared from the European mainland. This would be a parallel case to *Myrsidea sylviae*, which appears to be much more common on the Azores than on the mainland.

Unfortunately, almost all species in the *S. simplex* group are poorly described, and illustrations have been published only for a handful. Therefore, it is not presently possible to establish whether our specimens can be referred to a known species, or if they represent a new species. Adequate morphological revision of this species group, including type specimens of each species, is needed before it is possible to determine the origin and status of these specimens.

### Patterns of association

Chewing louse host-switching may be caused by phoresy on louse-flies [6, 28, 30]. This phenomenon is well known for *O. avicularia* [30], but less common for the smaller-bodied louse-flies of the genus *Ornithoica* [36]. To date, only one record of phoresy of lice on *O. turdi* has been published [40]. Lice belonging to the *Brueelia*-complex, such as *Guimaraesiella* and *Sturnidoecus* frequently use phoresy to colonise new hosts [3, 6, 30]. We recorded phoresy of one female of *G. amsel* on *O. turdi* and documented several cases of the occurrence of *Guimaraesiella* lice on atypical hosts. We cannot entirely exclude the possibility of accidental transmission of these lice during handling or through the bird bags, but due to circumstances such as the order in which birds were caught and handled, the occurrence of these lice on unusual hosts likely represents a natural but accidental infestation, most likely as a result of phoresy. Our results suggest that phoresy may be common on the Azores. We recorded phoresy and unusual louse-host associations related to phoresy only during April where louse-flies are not so abundant compared to September. On the other hand, due to their population dynamics chewing lice populations are known to grow in size during spring as a result of the onset of their host’s breeding period [73], which may be the reason why the interaction between chewing lice and louse-flies can be observed more often during this period.

From the view of co-occurrence of chewing lice, it is known that most passerines usually harbour at least one ischnoceran and one amblyceran species of louse [57]. Recorded co-occurrence of two species of ischnoceran lice is also well-documented thanks to microhabitat preference of different groups of these lice, i.e. recorded pairs *Turdinirmus*+*Sturnidoecus* and *Guimaraesiella+Philopterus* represent pairs of “body louse species”+“head louse species” [7]. These louse-genus pairs were all found on *T. merula*, the bird species with the highest diversity of ectoparasites recorded in our study; this is also the largest-bodied passerine bird we examined. This example is in accordance with previous studies that have demonstrated correlations between parasite species richness and host body size [38, 67].

### Patterns of distribution

We documented a relatively small fraction of the known diversity of louse-host associations for each of the eight examined passerine birds on the Azores (see Supplementary Table S1). This is in accordance with the data published by Rodrigues et al. [63], in which a lower species richness of feather mites was documented in Azorean passerines compared to on the European mainland. One striking example of this difference between the Azores and the mainland is the lack of lice of any species on any of the 30 examined specimens of *Serinus canaria*. To date, there are no records of lice from wild canaries; however, two species of lice, *Menacanthus eurysternus* and *Myrsidea serini* (Séguy, 1944), are frequently reported from captive canaries [31, 45, 47, 53, 55].

Lice reported from captive populations of *S. canaria* may derive from stragglers or contaminations among captive birds kept in close proximity. For instance, *M. serini* is also known to parasitize other fringillid and emberizid hosts [57], including *S. serinus* (Linnaeus, 1766), which is the closest relative of *S. canaria* [76]. Under artificial circumstances such as the pet trade, transmission between closely related hosts may be enough to explain the presence of *M. serini* in captive populations, even though it is absent in wild populations. However, such explanations cannot easily be extended to the other hosts we sampled, as multiple louse species frequently recorded from wild populations on the mainland were absent in our samples.

Colonisation of the Azores by passerines is of relative recent origin, while the ancestor of wild canary colonised the Azores Islands in the last 0.7–3 million years [2, 21]. *Turdus merula* colonised the islands in two consecutive events: first around 0.47 Mya and then more recently, approximately 0.09 Mya during the Pleistocene [64], similar to other species, such as *S. atricapilla* [22], *E. rubecula*, *F. coelebs* and *R. regulus* [60–62]. Thus, the Azores may have acted as a refugium of these birds during the Pleistocene, which certainly also affected their parasitic fauna.

Differences between the Azorean parasite fauna and that of the mainland may be due to sorting events such as “missing the boat” [52], also known as the “parasite island syndrome” [34, 49], in which certain parasites were absent on hosts that originally colonised the Azores, and thus are absent in their descendants. An alternative hypothesis may be that differences in ambient climate between the Azores and the mainland may have driven local populations of parasites to extinction after colonisation, a process called “drowning on arrival” [52], or “lost overboard” [41]. In this scenario, a parasite species may have been present among the original host individuals colonising an island, but whereas the hosts successfully established a new population, the parasites failed to do so, perhaps due to low prevalence, and thus became extinct [63]. This process may be impossible to differentiate from “missing the boat” events.

Climate-related differences in louse fauna composition on the same host in different parts of its range have been suggested for several groups of birds [25, 42], and may play a factor in the depauperate louse fauna of the Azores. In a study of the composition of the chewing louse fauna on scrub-jays *Aphelocoma californica* (Vigors, 1839) in the American southwest, Bush et al. [11] found that the genera *Myrsidea* and *Brueelia* appear to be affected differently by ambient humidity, whereas *Philopterus* appeared to be unaffected by ambient humidity. Similarly, Takano et al. [75] found that *Guimaraesiella* occurred only in more humid areas, whereas *Brueelia* occurred mainly in drier areas, and Carrillo et al. [13] found both *Brueelia* and *Philopterus* to be largely unaffected by arid environments.

Interestingly, our data from the Azores are not straight-forward with regards to the possible interaction between ambient humidity and the presence of lice. No host sampled on the Azores was found to be parasitized by all species of lice known from mainland Europe (Supplementary Table S1). However, collectively these hosts were parasitized by representatives of all common genera of chewing lice found on passerine hosts in Eurasia (genera *Brueelia, Guimaraesiella, Menacanthus, Myrsidea, Philopterus, Sturnidoecus*), as well as the less common thrush-specific genus *Turdinirmus*. Of the genera of lice known from the studied hosts in mainland Eurasia, only *Penenirmus* and *Rostrinirmus* are absent in our samples from the Azores. Thus, the supposedly “humid-adapted” *Myrsidea* and *Guimaraesiella*, the “arid-adapted” *Brueelia*, and the “humidity-indifferent” *Philopterus* were all found on the Azores, albeit not on the same host species. This may indicate that ambient humidity has little effect on what louse species occur on the Azores; however, other environmental factors may have influenced the louse fauna of this archipelago. Moreover, Malenke et al. [42] showed that differences in humidity preferences may occur within the same louse genus, suggesting that more detailed studies are needed to determine whether humidity has an effect on the lice collected from the Azorean birds.

There also seems to be no bias in what ecomorphs of lice are found on the Azores. Of the eight ischnoceran species we recorded, three (*Philopterus* and *Sturnidoecus*) belong to the “head louse” ecomorph, whereas the others are sometimes considered “generalists” [29] but are in fact specialised on different areas of the hosts’ body plumage [7, 43]; the “wing louse” ecomorph generally does not occur on passerines. The three genera of amblyceran lice recorded do not fall into ecomorphs in the same way, but each of them represent a different morphological group of lice, and the amblyceran fauna in our samples covers both families that usually occur on passerine hosts (Ricinidae and Menoponidae).

The apparent absence of a systematic environmental bias against any louse group in our samples from the Azores, when all lice are considered together, may indicate that more species of lice may be recovered with larger sample sizes, or that absences are due to random effects. More research focusing on a larger number of hosts and on other islands of the Archipelago may be necessary to determine whether some parasite species are truly absent, and if so why. Comparisons with the louse fauna of birds on other archipelagos would also be useful, particularly if comparisons could be made between islands that are relatively close to the mainland (e.g. the Canary Islands, Cape Verde, the Balearic Islands) and those that are more isolated (e.g. the Azores, Tristan de Cunha, Bermuda). Comparisons among islands or island groups of different size may also be valuable, as Bush et al. [12] found a correlation between habitat size and louse biodiversity. Such studies could, in turn, give important insights into what processes structure the biogeographical range of chewing lice and parasites in general.

## Supplementary material

Supplementary material is available at https://www.parasite-journal.org/10.1051/parasite/2020063/olm
*Table S1*. List of chewing lice for eight passerine birds examined in the Azores: all known parasite-host associations compared with those recorded in the Azores
*Table S2*. Proportion of birds (%) with a particular category of infestation of all chewing lice species combined on all parasitized passerine birds (*n* = 91), and also separately for the dominant species *Menacanthus eurysternus* on parasitized *Fringilla coelebs* (*n* = 30) in the Azores

## Conflict of interest

The authors declare that they do not have any conflict of interest.
