# Supplementary material for: Insect ectoparasites from wild passerine birds in the Azores Islands
Source: Parasite. 2020 Nov 20;27:64. doi: 10.1051/parasite/2020063 (PMC7680507; doi:10.1051/parasite/2020063)
Supplement: Supplementary file 1 — Table S1. List of chewing lice for eight passerine birds examined in the Azores: all known parasite-host associations compared with those recorded in the Azores Table S2. Proportion of birds (%) with a particular category of infestation of all chewing lice species combined on all parasitized passerine birds (n = 91), and also separately for the dominant species Menacanthus eurysternus on parasitized Fringilla coelebs (n = 30) in the Azores [file parasite-27-64-s1.pdf]

Supplementary Table S1. List of chewing lice for eight passerine birds examined in the Azores: all known parasite-host associations compared with those recorded in the Azores.

All known species	Species recorded in the Azores
**Family Fringillidae**	**Family Fringillidae**
***Carduelis carduelis* (Linnaeus, 1758)**	
Menacanthus alaudae (Schrank, 1776)	
Myrsidea serini (Séguy, 1944)	
Ricinus fringillae De Geer, 1778	
Brueelia densilimba (Nitzsch [In Giebel], 1866)	
Sturnidoecus blandus Zlotorzycka, 1964	
***Fringilla coelebs* Linnaeus, 1758**	
Menacanthus eurysternus (Burmeister, 1838)	*Menacanthus eurysternus* (Burmeister, 1838)
Myrsidea lyali Klockenhoff, 1984	
Ricinus fringillae De Geer, 1778	
Brueelia kluzi Balát, 1955	*Brueelia kluzi* Balát, 1955
Penenirmus serrahnensis Balát, 1982	
Philopterus fortunatus (Zlotorzycka, 1964)	
***Serinus canaria* (Linnaeus, 1758)**	
Menacanthus eurysternus (Burmeister, 1838)	
Myrsidea serini (Séguy, 1944)	
**Family Muscicapidae**	
***Erithacus rubecula* (Linnaeus, 1758)**	
Menacanthus eurysternus (Burmeister, 1838)	
Ricinus rubeculae (Schrank, 1776)	*Ricinus rubeculae* (Schrank, 1776)
Guimaraesiella tristis (Giebel, 1874)	*Guimaraesiella tristis* (Giebel, 1874)
Philopterus rubeculae (Denny, 1842)	
**Family Passeridae**	
***Passer domesticus* (Linnaeus, 1758)**	
Menacanthus eurysternus (Burmeister, 1838)	
Myrsidea quadrifasciata (Piaget, 1880)	
Ricinus fringillae De Geer, 1778	
Brueelia cyclothorax (Burmeister, 1838)	
Philopterus fringillae (Scopoli, 1772)	
Rostrinirmus boeevi (Balát, 1958)	

Rostrinirmus ruficeps (Nitzsch [In Giebel], 1866)	
**Family Regulidae**	
***Regulus regulus* (Linnaeus, 1758)**	
Ricinus frenatus (Burmeister, 1838)	
Philopterus gustafssoni Najer et al., 2020	*Philopterus gustafssoni* Najer et al., 2020
**Family Sylviidae**	
***Sylvia atricapilla* (Linnaeus, 1758)**	
Menacanthus curuccae (Schrank, 1776)	
Menacanthus eurysternus (Burmeister, 1838)	
Myrsidea sylviae Sychra & Literak, 2008	*Myrsidea sylviae* Sychra & Literak, 2008
Brueelia atricapillae (Soler Cruz, Alcántara Ibañez & Florido-Navío, 1984)	
Guimaraesiella tovornikae (Balát, 1981)	*Guimaraesiella tovornikae* (Balát, 1981)
**Family Turdidae**	
***Turdus merula* Linnaeus, 1758**	
Menacanthus eurysternus (Burmeister, 1838)	*Menacanthus eurysternus* (Burmeister, 1838)
Myrsidea thoracica (Giebel, 1874)	
Ricinus elongatus (Olfers, 1816)	
Brueelia jacobi Eichler, 1951	
Guimaraesiella amsel (Eichler, 1951)	*Guimaraesiella amsel* (Eichler, 1951)
Philopterus turdi (Denny, 1842)	*Philopterus turdi* (Denny, 1842)
Turdinirmus merulensis (Denny, 1842)	*Turdinirmus merulensis* (Denny, 1842)
	Sturnidoecus sp.

Supplementary Table S2. Proportion of birds (%) with a particular category of infestation of all chewing lice species combined on all parasitized passerine birds (n = 91), and also separately for the dominant species *Menacanthus eurysternus* on parasitized *Fringilla coelebs* (n = 30) in the Azores.

	All chewing lice species on all host species	*M. eurysternus* on *Fringilla coelebs*
very light infestation (1–10 lice/host)	69.2	59.9
light infestation (11–20 lice/host)	11.0	10.0
medium infestation (21–30 lice/host)	6.6	10.0
heavy infestation (31–50 lice/host)	4.4	6.7
very heavy infestation (51–100 lice/host)	6.6	6.7
extremely heavy infestation (>100 lice/host)	2.2	6.7
